# Stereoselectivity of Isoflurane in Adhesion Molecule Leukocyte Function-Associated Antigen-1

**DOI:** 10.1371/journal.pone.0096649

**Published:** 2014-05-06

**Authors:** Weiming Bu, Luis M. Pereira, Roderic G. Eckenhoff, Koichi Yuki

**Affiliations:** 1 Department of Anesthesiology and Critical Care, Perelman School of Medicine, University of Pennsylvania, Philadelphia, Pennsylvania, United States of America; 2 Department of Anesthesiology, Perioperative and Pain Medicine, Boston Children's Hospital, Boston, Massachusetts, United States of America; 3 Department of Anaesthesia, Harvard Medical School, Boston, Massachusetts, United States of America; Massachusetts General Hospitalm, United States of America

## Abstract

**Background:**

Isoflurane in clinical use is a racemate of S- and R-isoflurane. Previous studies have demonstrated that the effects of S-isoflurane on relevant anesthetic targets might be modestly stronger (less than 2-fold) than R-isoflurane. The X-ray crystallographic structure of the immunological target, leukocyte function-associated antigen-1 (LFA-1) with racemic isoflurane suggested that only S-isoflurane bound specifically to this protein. If so, the use of specific isoflurane enantiomers may have advantage in the surgical settings where a wide range of inflammatory responses is expected to occur. Here, we have further tested the hypothesis that isoflurane enantioselectivity is apparent in solution binding and functional studies.

**Methods:**

First, binding of isoflurane enantiomers to LFA-1 was studied using 1-aminoanthracene (1-AMA) displacement assays. The binding site of each enantiomer on LFA-1 was studied using the docking program *GLIDE*. Functional studies employed the flow-cytometry based ICAM binding assay.

**Results:**

Both enantiomers decreased 1-AMA fluorescence signal (at 520 nm), indicating that both competed with 1-AMA and bound to the αL I domain. The docking simulation demonstrated that both enantiomers bound to the LFA-1 “lovastatin site.” ICAM binding assays showed that S-isoflurane inhibited more potently than R-isoflurane, consistent with the result of 1-AMA competition assay.

**Conclusions:**

In contrast with the x-ray crystallography, both enantiomers bound to and inhibited LFA-1. S-isoflurane showed slight preference over R-isoflurane.

## Introduction

Many biologically active molecules, including drugs, exist in different chiral forms, and it is possible that each specific enantiomer can interact with their targets differently, thereby potentially eliciting different biological responses. Thus, the potential exists for a racemic mixture of a compound to cause diverse, and perhaps unwanted, side effects. Regardless, racemic drugs are used commonly in clinical medicine, as the purification or synthesis of one specific enantiomer can be costly. Novel drug discovery can be even more costly, so there has been recent interest in exploiting enantioselectivity in the last two decades. While the majority of drugs on the market were sold as racemic mixtures in early 1990s, about 40% of drugs were marketed as single enantiomers by 2002 [1]. Some of these single enantiomers were newly developed, while some were isolated from the previously marketed racemic mixtures [2]. The advantages of using specific enantiomers over the racemic mixture might include their less complex and more selective pharmacodynamic profiles, and the potential for improved therapeutic index and the reduction of side effects. In extreme cases, one enantiomer may act as an antagonist (such as R(-)-PN 202-791; calcium channel antagonist), while the other may be an agonist (such as S(+)-PN 202-791; calcium channel agonist) [3].

The interest in single enantiomers has also reached the anesthesia field, as demonstrated by the introduction of ropivacaine and levo-bupivacaine [3]. Further, even the inhalational general anesthetics have chiral centers. For example, isoflurane has been successfully separated into its enantiomers by Huang et al. and others in 1990s [4], allowing investigators to test the potency of each separately. The S- enantiomer of isoflurane was shown to be approximately 2-times more potent *in vitro* and *in vivo* than R- enantiomer isoflurane (**Table 1**). However, the structural basis for the differential potency of isoflurane enantiomers has yet to emerge, but it has been reported that S-isoflurane binds with higher affinity to model proteins, like human serum albumin [5].

**Table 1:** The previous studies using isoflurane enantiomers.

Type of experiment	Study results	Reference
*In vivo*	The lipid emulsion of isoflurane injection to rats. S(+) was 40+/−8% more potent than R(−) at producing loss of righting reflex.	[35]
*In vivo*	The inhalation of isoflurane enantiomers to rats. Minimum alveolar concentMAC was S(+); 1.06%, R(−); 1.62%, suggesting S(+) was about 50% more potent.	[36]
*In vivo*	The determination of MAC using rats. Inhalational route. S(+): 0.0144 +/− 0.0012 atm, R(−); 0.0169 +/− 0.0020 atm. Not statistically significant.	[37]
*In vivo*	Intraperitoneal injection of isoflurane enantiomers to mice. Sleep time was longer with S(+) over R(−).	[38]
*In vitro*	S(+) was more potent and efficacious than R(−) in enhancing [^3^H] flunitrazepam binding to GABA_A_ receptor complex.	[13]
*In vitro*	S(+) had greater increase in GABA receptor mediated IPSCs than R(−)	[14]
*In vitro*	S(+) was 1.6 times as potent as R(−) in augmenting GABA gated Cl flux.	[15]
*In vitro*	Cl flux induced by GABA was potentiated by isoflurane. The maximum stereoselectivity occurred at 0.3 mM isoflurane (S > R), about 2 times.	[16]
In *vitro*	No stereospecific effects of isoflurane in isolated guinea pig hearts (the effect on LVP, AV conduction, coronary flow)	[48]
*In vitro*	No stereoselectivity of isoflurane seen to inhibit isradipine binding to L-type calcium channel	[49]
*In vitro*	S(+) bound bovine serum albumin with slower association and dissociation rates than R(−). No difference in static condition.	[50]
*In vitro*	S(+) was twofold more effective than R(−) both in eliciting the anesthetic-activated potassium current and in inhibiting a current mediated by neuronal nicotinic acetylcholine receptors	[17]

The reported studies of isoflurane enantiomers are summarized in the table.

MAC; minimum alveolar concentration, GABA; gamma-aminobutyric acid, LVP; left ventricular pressure, AV; atrio-ventricular, IPSC; inhibitory postsynaptic current.

**Figure 1:**
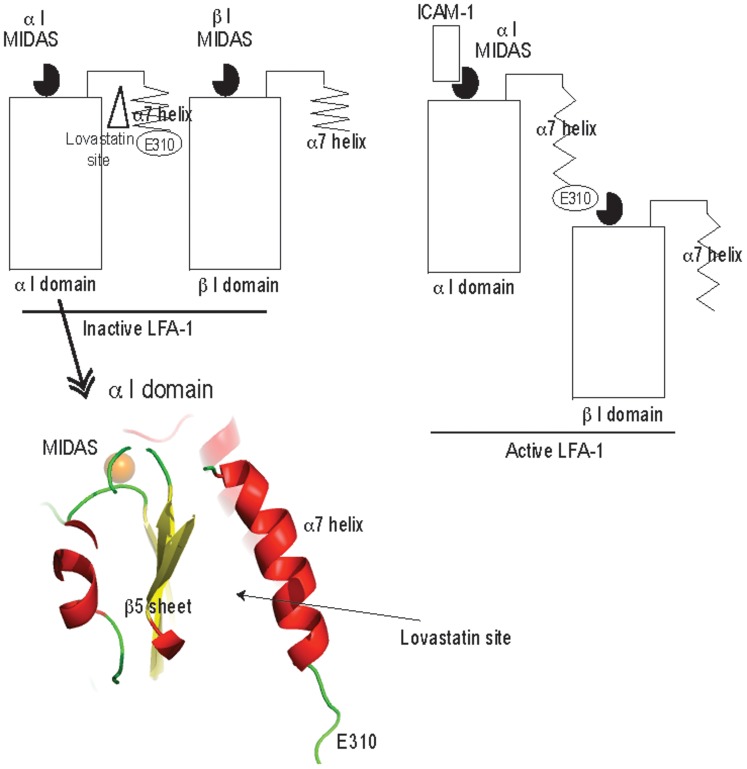
Schematic description of leukocyte function-associated antigen-1 (LFA-1) structure and “lovastatin site”. Inactive and active forms of LFA-1 are shown in cartoon. The α7 helix is very flexible as shown. In an inactive form, there is a cavity called “lovastatin site” underneath the α7 helix of the αL I domain. There is no active signal communication from the βI domain to the αI domain. However, in an active form, the α7 helix of the αI domain gets unwound and Glu-310, which has a negative charge, interacts with metal-ion dependent adhesion site (MIDAS) (positive charge) in the βI domain. This is how the βI domain communicates with the αI domain. And MIDAS in the αI domain binds to the ligand.

Previously we demonstrated that isoflurane interacted with the adhesion molecule leukocyte adhesion-associated antigen-1 (LFA-1). This protein is expressed ubiquitously on leukocytes and is involved in various immunological actions such as leukocyte arrest on the endothelium [6] and immunological synapse formation [7]. This heterodimeric molecule consisting of α- and -β subunits is activated through the intracellular activation signals called the “inside-out” signal and undergoes dynamic conformational changes [8], [9]. The α7 helix of the ligand binding domain (the αL I domain) of the α subunit is extremely flexible [10]. In a resting (inactive) state, the α7 helix remains helical, forming a pocket called the “lovastatin site.” In a high-affinity (active) state, however, an unwinding, downward movement of the α7 helix occurs causing a loss of the “lovastatin site”, which allows the αL I domain to bind ligands at the metal-ion dependent adhesion site (MIDAS) (**Figure 1**). The X-ray crystallization of racemic isoflurane complexed with the αL I domain demonstrated that isoflurane bound to this “lovastatin site”[11], and the functional relevance of this binding in LFA-1 blockade by isoflurane was shown by the mutagenesis experiment [12]. Interestingly, further refinement of this complex suggested that only S-isoflurane, fit into the electron density map of this region; the R-isomer produced a steric clash with lining residues. This seemingly extreme example of isoflurane stereoselectivity on LFA-1 suggested by the X-ray crystallographic structure is striking as compared to the modest, functional stereoselectivity suggested by its central nervous system effects [13], [14], [15], [16], [17]. Our previous study demonsrated that the interaction of racemic isoflurane with LFA-1 had functional relevance *in vivo* [18]. Thus, if the suggested selectivity on LFA-1 can be demonstrated in solution, it may open the door to more selective application of these drugs in the perioperative setting.

In this study, we tested the hypothesis that the enantioselectivity of isoflurane suggested by the crystal structures of LFA-1-isoflurane complex, is also observed in solution binding and functional studies.

## Methods

### Protein expression and purification

The αL I domain wild type (WT, residues 128-307 of the integrin αL subunit) (low affinity conformer) of LFA-1 were expressed in BL21 (DE3) cells. The αI domains were expressed as inclusion bodies, which were solubilized and refolded as previously described [19]. Soluble full ectodomain LFA-1 protein was expressed from CHO Lec 3.2.8.1 cells stably transfected with LFA-1 plasmids and purified as previously described [20].

### Cell-free LFA-1: ICAM-1 binding assays

LFA-1: ICAM-1 binding assays were performed as previously described with minor modifications [21]. Briefly, soluble LFA-1 protein (10 µg/mL) was immobilized on the capturing antibody named anti-Velcro antibody (Immune Disease Institute, Boston, MA) on 96 well plates. Following blocking, human ICAM-1-Fcα fusion protein (5 µg/mL) was added to wells with HEPES-buffered saline (HBS) containing 1 mM MnCl_2_ and in some cases, 10-25 µM 1-aminoanthracene (1-AMA) (Sigma; St. Louis, MO, USA). After incubation for 1 hour at room temperature, unbound ICAM-1 was washed off and bound ICAM-1 was detected using peroxidase-labeled goat anti-human IgA and substrate (BD, Franklin Lakes, NJ, USA). Absorbance was measured at 405 nm. ICAM-1 binding % was defined as [(optimal density (OD) of 1-AMA]/OD of mock-treated sample] × 100%.

### Transient transfection of LFA-1 in 293 T cells

293 T cells were cultured in HEPES modified - Dulbecco's modified Eagle medium (DMEM)/10% fetal bovine serum (FBS) at 37°C, 5% CO_2_. Wild type LFA-1 plasmid was transfected using Lipofectamine 2000 (Invitrogen; Carlsbad, CA, USA).

### Fluorescence microscopy

293 T cells were transiently transfected with LFA-1 on glass bottom dishes (MatTek Corporation; Ashland, MA, USA). LFA-1 on the cells was stained with TS1/12 antibody (Immune Disease Institute, Boston, MA, USA) followed by anti-mouse Cy5 antibody (Life Technologies; Grand Island, NY, USA). 1-AMA was added to medium, incubated for 10 minutes, and then the cells were examined with fluorescence microscopy.

### Chiral gas chromatography

Isoflurane enantiomers (S-isoflurane and R-isoflurane) were kindly provided by Baxter (Cambridge, MA, USA). Racemic isoflurane was from Abbott Laboratories (Abbott Park, IL, USA). We first used chiral gas chromatography with mass spectrometry detection (GC/MS) to evaluate the purity of each optical enantiomer. Samples of isoflurane were diluted in heptane (Sigma) and injected into the gas chromatography (Model G1540N-6410N, Agilent Technologies; Santa Clara, CA, USA). We used a 30-m, 0.25-mm, 0.25 µm 20% permethylated β-cyclodextrin chiral 20B column (Agilent Technologies). The parameters were a split ratio of 1: 20, injector temperature of 70m°C, oven temperature of 60°C, and carrier gas hydrogen at 1 mL/min. Mass spectrometry quantification was conducted in single ion model at m/z 51, 117 and 141.

### 1-AMA competition assay

The competition of 1-AMA binding with the isoflurane enantiomers was performed using the αL I domain. The αL I domain (0.2 µM) was first pre-equilibrated with 10 µM 1-AMA (Sigma; St. Louis, MO, USA). After addition of 0.1 µM to 4 mM of S-, or R- isoflurane, samples were excited at 380 nm and emission spectra were collected from the range of 400 nm to 700 nm. The reduction of the fluorescence signal at 540 nm was corrected by subtracting the baseline fluorescence curves of 1-AMA and the αL I domain, and plotted against isoflurane enantiomer concentration. Both competition curves were fitted to variable slope Hill curves. Analysis was performed using PRISM 5 software (Graph Pad Software, La Jolla, CA, USA).

### Docking simulation of S- and R-isoflurane

The structure of the αL I domain was obtained from the Protein Data Bank (PDB) 1ZOO [22]. The program *GLIDE* (Schrodinger; Cambridge, MA, USA) was used to perform rigid molecular docking of isoflurane enantiomers with the αL I domain, as previously described [20]. The structures of the isoflurane enantiomers were obtained through PubChem (http://pubchem.ncbi.nlm.nih.gov/). Isoflurane binding position was sought with the grid size of 25 × 25 × 25 Å^3^ and the centroid grid residue of Tyr-257. No positional constraint was applied. *GLIDE* has a scoring system (“glidescore”) that ranks docked pairs based on the predicted interaction free-energy. The pair with the most negative glidescore is considered to have the highest affinity, thus we selected the docked pair with the most negative glidescore.

### Cell-based Intercellular adhesion molecule-1 (ICAM-1) binding assay

ICAM-1 is the major endogenous ligand for LFA-1. A cell-based LFA-1: ICAM-1 binding assay was performed using flow cytometry as previously described [23]. Briefly, 293 T cells transiently transfected with LFA-1 were harvested in HBS containing 10 mM EDTA 48 hours after transfection. Cells were washed with HBS, and then resuspended in HBS. Cells were aliquoted to polymerase chain reaction tubes (Axygen; Union City, CA, USA) and then centrifuged. Cell pellets were given150 µl aliquot of HBS, 2 mM MnCl_2_ containing isoflurane at 2 × final concentrations, and another 150 µl aliquot of HBS containing 10 µg/mL ICAM-1-Fcα fusion protein. Tubes were immediately capped, mixed, and incubated for 30 minutes at room temperature. Following wash, cells were incubated with anti-human IgA-FITC (Invitrogen) as a secondary antibody for 30 minutes. The cells were then washed and subjected to flow cytometry analysis using a FACScan instrument (BD Bioscience; San Jose, CA, USA). ICAM-1 binding % was defined as [mean fluorescence intensity (MFI) of samples at various concentrations of isoflurane divided by mean fluorescence intensity of mock-treated sample] × 100%.

### Statistical analysis

All the statistical analyses were performed using PRISM 5 software. The details of statistical analysis were described in the corresponding figure legends. P<0.05 was considered statistically significant.

## Results

### The interaction of 1-AMA with LFA-1

**Figure 2:**
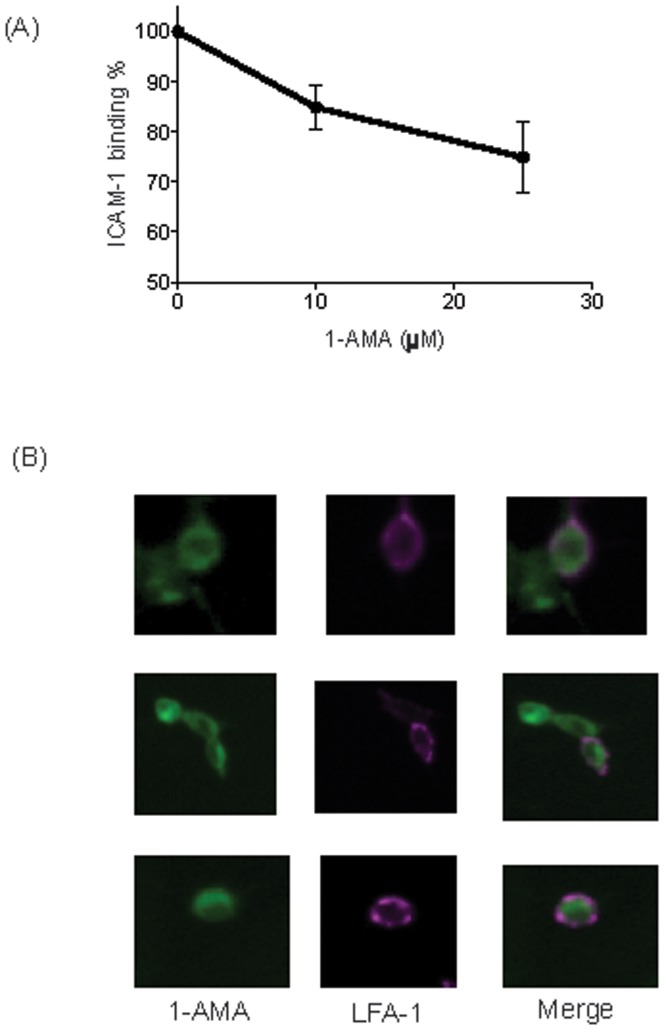
The interaction of 1-aminoanthracene (1-AMA) with leukocyte function-associated antigen-1 (LFA-1). (A) LFA-1: ICAM-1 binding was tested in cell-free system in the presence of 1-AMA. 1-AMA impaired ICAM-1 binding. (B) Localization of 1-AMA was tested in transient LFA- 1 transfectants. In Figure, green represents 1-AMA, and red represents LFA-1.

1-AMA is a small molecule with environment-dependent fluorescence and general anesthetic properties as demonstrated by the potentiation of gamma-aminobutyric acid (GABA)-ergic transmission [24], [25], [26]. It has been used to explore protein cavities, and binds to a well-characterized “general anesthetic site” in horse spleen apoferritin (HSAF) [26], [27]. We previously demonstrated that 1-AMA exhibited fluorescence shift in the presence of αL I domain, suggesting that 1-AMA interacted with LFA-1 [21]. Our previous direct photolabeling work showed that propofol bound the “lovastatin site.” In addition propofol displaced 1-AMA from LFA-1. We explored the size of available cavities on the surface of αL I domain and found that only the “lovastatin site” was a larger cavity than 1-AMA. Thus we concluded that 1-AMA bound the “lovastatin site.” This site is also the binding site of LFA-1 allosteric antagonists, and therefore we hypothesized that 1-AMA would block LFA-1. As shown in **Figure 2A**, 1-AMA inhibited the binding of LFA-1 to its ligand ICAM-1.

### The binding of 1-AMA to LFA-1 on the cell surface

The interaction of 1-AMA with LFA-1 was studied in a cell-based system as well. Using microscopy, we asked if 1-AMA binds to LFA-1 on the surface of transfected cells. 1-AMA fluorescence was clearly co-localized with LFA-1 (**Figure 2B**), however, it was also seen in non-transfected cells, indicating that it has other binding targets. Because there are many proteins with hydrophobic cavities that can accommodate 1-AMA (∼500 Å^3^)[28], this was predicted. Our previous work showed that isoflurane bound LFA-1 at the “lovastatin site” [12], [20], so we hypothesized that isoflurane and 1-AMA would compete at this site, with the S-enantiomer being more potent than R-isoflurane. Because 1-AMA displacement requires a small amount of reagents and the available amount of isoflurane enantiomers is limited, we opted to use this method in a cell-free system.

### The purity of S- and R- isoflurane enantiomers

We validated that the S- and R-enantiomers as supplied by the company were greater than 99% pure, and that the racemic mixture consisted of 50% (±2%) of each. (**Figure S1A-C**).

### Interactions of the αL I domain with two isoflurane enantiomers

**Figure 3:**
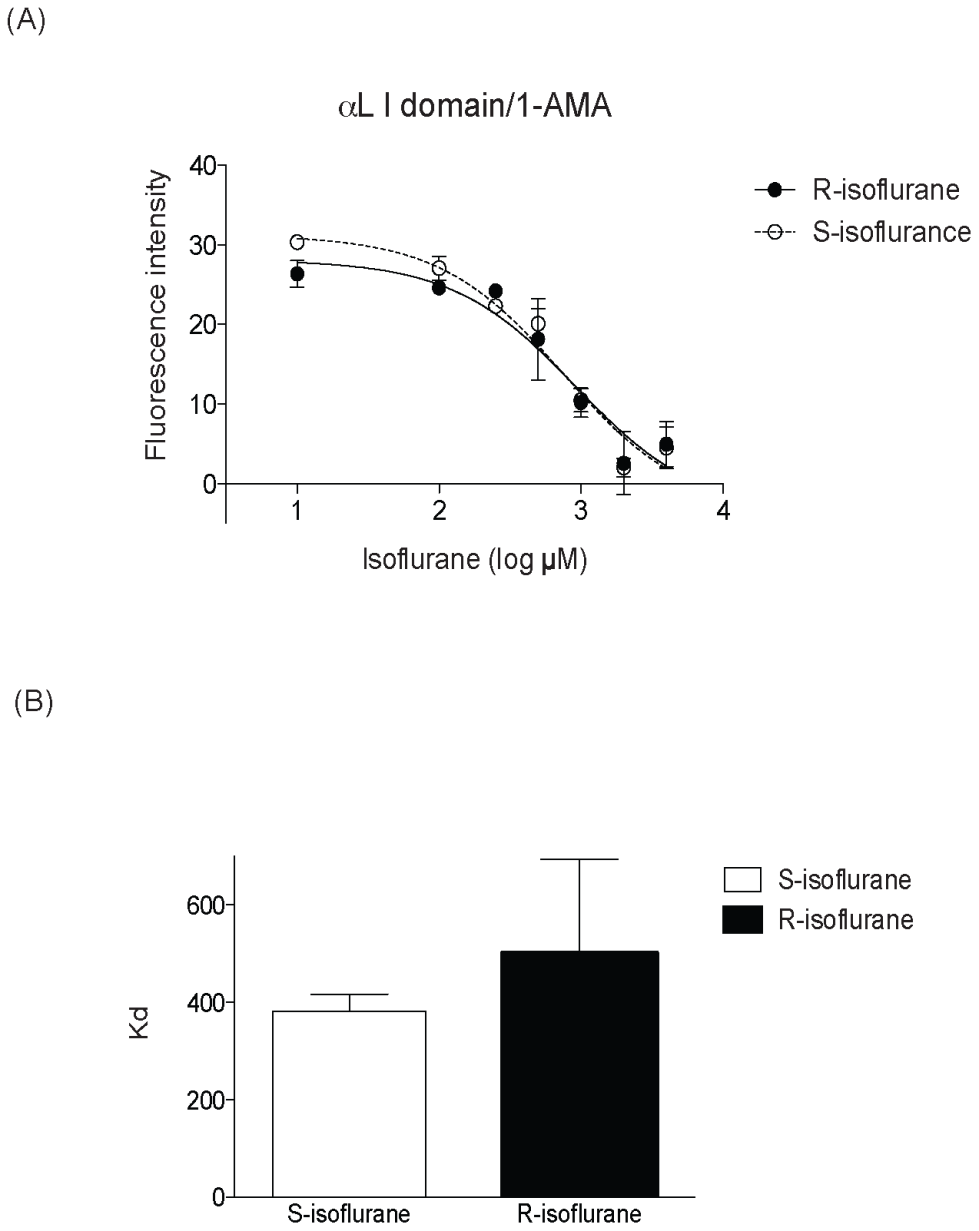
The determination of isoflurane enantiomers' binding to the αL I domain using 1-aminoanthracene (1-AMA). (A) The reduction of fluorescence intensity versus isoflurane concentration after displacement of 1-AMA from the αL I domain. Error bars indicate standard deviation (S.D.) of means of three independent experiments. (B) First, Kd values of S- and R-isoflurane in each independent experiment was obtained. Then the average Kd values of S- and R-isoflurane of three independet experiments were calculated (S-isoflurane 374.8 µM, R-isoflurane 460.2 µM). Data represent mean +/- S.D. F test was used for statistical analysis. No statistical significance was observed (p = 0.0629).

Our hypothesis was that only S-isoflurane binds to the “lovastatin site”. In contrast, however, *both* enantiomers reduced fluorescence intensity, suggesting that they both interact with the lovastatin site on the αL I domain (**Figure 3A**). The dissociation constants (Kd) for S-isoflurane and R-isoflurane (374.8 µM and 460.2 µM, resp.) were not statistically different (**Figure 3B**).

### Docking of S- and R-isoflurane into the αL I domain

**Figure 4:**
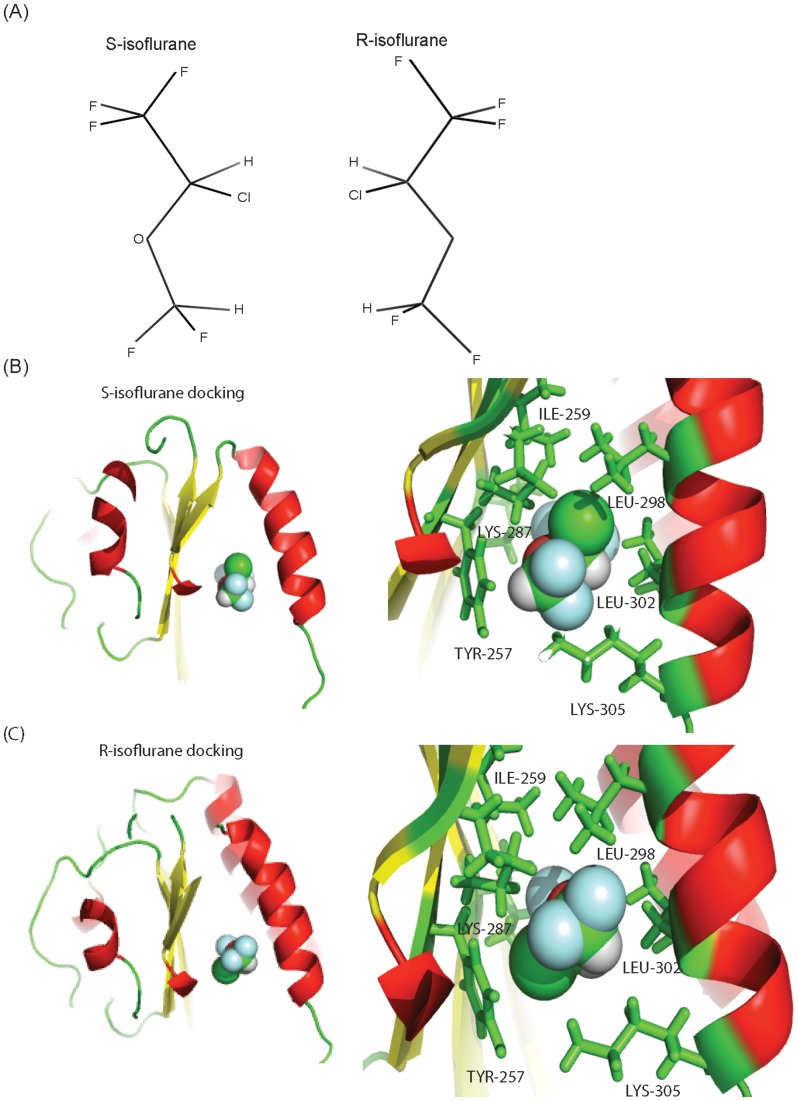
The docking of isoflurane enatiomers at “lovastatin site”. (A) The scheme of S- and R-isoflurane is shown. (B, C) The docking result of isoflurane enatiomers onto the αL I domain is shown. Right panels showed blowup of the docking site. A part of amino acid residues within 4 Å^3^ from isoflurane were shown with side chains in green. In isoflurane: red, oxygen; green, chloride; light blue, fluoride.

Both enantiomers were successfully docked to the “lovastatin site” using GLIDE (**Figure 4B and 4C** **)**. The neighboring amino acid residues are listed in **Table 2**. In both S-and R- isoflurane docking models, the trifluoromethyl heads were in the same orientation and formed hydrophobic interactions with Ile-235 and Leu-302. However, the difluoromethyl groups assumed different orientations. The difluoromethyl group in R-isoflurane had hydrophobic interactions with Leu-298 and Leu-302, while in S-isoflurane it interacted with Tyr-257 and Leu-302. Consistent with the 1-AMA experiments, the comparison of the docking scores indicated an inability to detect a significant difference of affinity to the αL I domain between S- and R-isoflurane (**Table 3**).

**Table 2:** The distance of near-by amino acid residues from isoflurane (< 4 Å).

Amino acid	R-isoflurane (Å)	S-isoflurane (Å)
Leu-132	3.0	3.2
Phe-134	3.9	3.6
Phe-153	3.4	2.8
Val-157	2.6	2.4
Ile-235	2.5	2.5
Tyr-257	3.9	2.9
Ile-259	3.5	3.6
Leu-198	2.8	3.5
Leu-302	2.4	2.4
Lys-305	3.8	3.9

**Table 3:** GLIDE docking score.

	S-isoflurane	R-isoflurane
GLIDE score	-4.495	-4.321

### Inhibition of LFA-1 by S- and R- isoflurane

**Figure 5:**
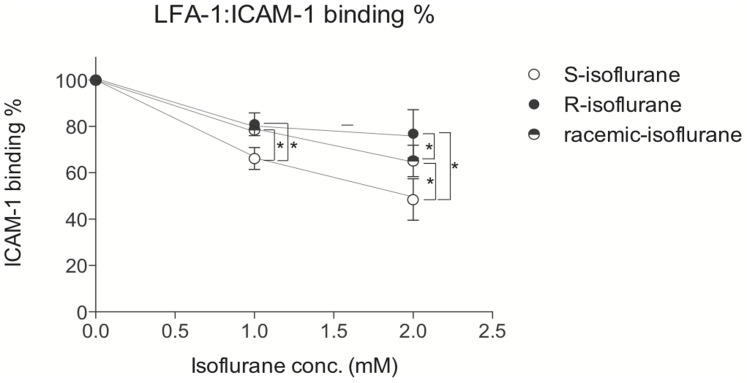
The ligand binding assay of leukocyte function-associated antigen-1 (LFA-1) using isoflurane enantiomers. The binding of LFA-1 to ICAM-1 in the presence of isoflurane (racemate, enantiomers) was tested using 293T cells overexpressing LFA-1 using flow cytometry as described in the method. Data represent mean +/- S.D. of triplicates. To compare any difference in ICAM-1 binding % in the presence of racemic, S- and R- isoflurane, statistical analysis was performed using two-way ANOVA with Bonferroni *post hoc* analysis. * denotes p<0.05.

The above results indicate that both isoflurane enantiomers bind to the “lovastatin site,” although subtle differences may exist which might be detectable using functional assays. Thus, we tested if both enantiomers could inhibit ligand (ICAM) binding. We used Mn^2+^ to activate LFA-1, which mimics inside-out signaling as described above. Also we confirmed that concentrations of S-, R- and racemic isoflurane remained steady during the incubation for 30 minutes. Both S- and R-isoflurane inhibited the binding of ICAM-1 to LFA-1 (**Figure 5**). Interestingly, S-isoflurane inhibited ICAM-1 binding ∼50% more potently than R-isoflurane, indicating the presence of functional enantioselectivity of isoflurane enantiomers to LFA-1.

## Discussion

Here we have demonstrated that both S- and R-isoflurane interact with LFA-1 at the lovastatin site, and impair its ligand binding. 1-AMA displacement assay and docking simulations were unable to detect a statistically significant difference in the interaction of the αL I domain with isoflurane enantiomers. Both enantiomers inhibited the binding of LFA-1 to ICAM-1 in cell-based assays, with the S-enantiomer being more potent. The enantioselectivity of isoflurane on anesthetic targets in the CNS has been documented as in **Table 1**, and in general indicate a very modest effect. This study is the first to investigate the enantioselectivity of targets relevant to immune function.

Our experimental results were inconsistent with structural analysis of previous x-ray crystallography experiments, which indicated that R-isoflurane could not bind this pocket due to steric clash with Tyr-257 [11]. While X ray crystallography is a very useful tool to provide detailed structural information, it may not reflect subtle changes in protein dynamics that allow accommodation of ligands (“induced fit”). Also, numerous lattice contacts at the protein-protein interfaces can subtly alter the crystal structure from the solution structure. Examples are alpha-lactalbumin [29], myoglobin [30] and aspartate transcarbamylase [31]. Finally, it is known that crystallization may not demonstrate structural variability as in the case of intestinal fatty-acid-binding apoprotein where X-ray crystallization did not demonstrate the variability of backbone structure [32]. Both 1-AMA displacement and cell-based ICAM-1 binding assays, were performed in a solution setting where dynamics should be retained. The “lovastatin site” underneath the C-terminal α7-helix was demonstrated to be the most variable region in reported I-domain X-ray crystallographic structures [33], as demonstrated by the difference of cavity size depending on the crystallization conditions [21]. The region was quite flexible in solution nuclear magnetic resonance structures [10]. Therefore, the probable explanation of the discrepancy between the structural and our data is a loss of normal protein flexibility or dynamics [10] that might normally result in “induced fit”, and which are reduced in the crystal due to multiple lattice contacts, leading into the altered features of small molecule binding sites. Our study further stresses the importance of solution-based assay for the validation of crystallographic studies.

Pfeiffer's rule states that the stereoselectivity of a drug is a function of the mass of drug necessary to produce its pharmacological effects [34]. Given that isoflurane's plasma concentration that produces general anesthetic effects is relatively high (∼ mM range), it is expected that isoflurane would demonstrate only modest stereoselectivity. Accordingly, previous *in vivo* studies of isoflurane enantiomers did not demonstrate more than a 20-50% difference in anesthetic effect [35], [36], [37], [38]. Our cell-based functional results showed that a similar magnitude of functional stereoselectivity was the case for LFA-1 as well. Previously, we demonstrated that isoflurane also bound the top domain of the β2 subunit called the βI domain, which is also an important regulator of ICAM-1 binding [20]. There could also be a difference of affinity against this domain between S- and R- isoflurane, which may explain the small difference of the results between 1-AMA displacement assay (or docking simulation) using only the αL I domain and cell-based ligand binding assay. Unfortunately, the fact that the isolated β2 I domain protein has not been successfully expressed [39] prevented us from testing this hypothesis.

LFA-1 antagonism by isoflurane has a few intriguing clinical implications. For example, LFA-1 knockout mice had a higher mortality than wild-type mice after intraperitoneal S. pneumoniae infection [40]. However, Emoto et al. demonstrated that LFA-1 knockout mice were *resistant* to lipopolysaccharide-induced liver injury [41]. Further, Miyamoto et al. showed that LFA-1 knockout mice had resistance to Listeria monocytogenes infection [42]. Blockade of LFA-1 has been shown to be beneficial in graft survival in various transplantation models [43], [44], [45]. A clinical pilot study of allo-islet transplantation recipients treated with the humanized anti-LFA-1 antibody efalizumab showed a favorable outcome [46]. In addition, efalizumab has been used to treat psoriasis [47]. Unfortunately, this drug has been withdrawn from the market due to a risk of fatal brain infections. These studies demonstrate that LFA-1 blockade could be beneficial or detrimental depending on circumstances. Also it has to be noted that many of these studies have been done with permanent LFA-1 blockade (knockout mice), or antibody blockade which usually has a prolonged effect. In our previous studies, we demonstrated that a short exposure of isoflurane (2–4 hours) attenuated neutrophil recruitment in the setting of skin inflammation, a process in which LFA-1 plays a large role. In the future, the impact of transient LFA-1 blockade by anesthetics needs to be examined. The answer to this question will allow us to consider to redesigning, or reformulating our anesthetic drugs to mitigate this LFA-1 functional alteration.

Additional finding in this study is that 1-AMA, which is a general anesthetic binding site probe, possessing general anesthetic activity, is an LFA-1 antagonist and bound LFA-1 on the cell surface. However, the existence of other targets is likely, which clouds interpretation.

In conclusion, we have demonstrated that both isoflurane enantiomers interact with LFA-1, an important component of immunologic cascades, with evidence for subtle enantioselectivity. The clinical significance of this interaction remains to be determined in the future.

## Supporting Information

Figure S1 **Chiral gas chromatography of isoflurane.** The gas chromatographic traces were shown. (A) racemic isoflurane, (B) S- or (C) R- isoflurane. The area under the curve was used to calculate the composition of S- and R-isoflurane in each solution. X-axis represents retention time (min), and y-axis represents detector (arbitrary unit).
(TIFF)

## References

[1] Shimazawa R, Nagai N, Toyoshima S, Okuda H (2008) Present state of new chiral drug development and review in Japan. Journal of Health Science 54: 23–29.

[2] Agranat I, Caner H, Caldwell J (2002) Putting chirality to work: the strategy of chiral switches. Nat Rev Drug Discov 1: 753–768. 12360254 10.1038/nrd915

[3] Nau C, Strichartz GR (2002) Drug chirality in anesthesia. Anesthesiology 97: 497–502. 12151942 10.1097/00000542-200208000-00029

[4] Huang CRL, Halpern D, Vernice G (1993) Preparation of the Isoflurane Enantiomers. J Org Chem 58: 7382–7387.

[5] Eckenhoff RG (1998) Do specific or nonspecific interactions with proteins underlie inhalational anesthetic action? Mol Pharmacol 54: 610–615. 9765502

[6] Shamri R, Grabovsky V, Gauguet JM, Feigelson S, Manevich E, et al (2005) Lymphocyte arrest requires instantaneous induction of an extended LFA-1 conformation mediated by endothelium-bound chemokines. Nat Immunol 6: 497–506. 15834409 10.1038/ni1194

[7] Springer TA, Dustin ML (2012) Integrin inside-out signaling and the immunological synapse. Curr Opin Cell Biol 24: 107–115. 22129583 10.1016/j.ceb.2011.10.004 PMC3294052

[8] Qin J, Vinogradova O, Plow EF (2004) Integrin bidirectional signaling: a molecular view. PLoS Biol 2: e169. 15208721 10.1371/journal.pbio.0020169 PMC423143

[9] Nishida N, Xie C, Shimaoka M, Cheng Y, Walz T, et al (2006) Activation of leukocyte beta2 integrins by conversion from bent to extended conformations. Immunity 25: 583–594. 17045822 10.1016/j.immuni.2006.07.016

[10] Legge GB, Kriwacki RW, Chung J, Hommel U, Ramage P, et al (2000) NMR solution structure of the inserted domain of human leukocyte function associated antigen-1. J Mol Biol 295: 1251–1264. 10653701 10.1006/jmbi.1999.3409

[11] Zhang H, Astrof NS, Liu JH, Wang JH, Shimaoka M (2009) Crystal structure of isoflurane bound to integrin LFA-1 supports a unified mechanism of volatile anesthetic action in the immune and central nervous systems. FASEB J 23: 2735–2740. 19332643 10.1096/fj.09-129908 PMC2717780

[12] Yuki K, Astrof NS, Bracken C, Yoo R, Silkworth W, et al (2008) The volatile anesthetic isoflurane perturbs conformational activation of integrin LFA-1 by binding to the allosteric regulatory cavity. FASEB J 22: 4109–4116. 18708587 10.1096/fj.08-113324 PMC2614612

[13] Moody EJ, Harris BD, Skolnick P (1993) Stereospecific actions of the inhalation anesthetic isoflurane at the GABAA receptor complex. Brain Res 615: 101–106. 8395953 10.1016/0006-8993(93)91119-d

[14] Jones MV, Harrison NL (1993) Effects of volatile anesthetics on the kinetics of inhibitory postsynaptic currents in cultured rat hippocampal neurons. J Neurophysiol 70: 1339–1349. 7506753 10.1152/jn.1993.70.4.1339

[15] Quinlan JJ, Firestone S, Firestone LL (1995) Isoflurane's enhancement of chloride flux through rat brain gamma-aminobutyric acid type A receptors is stereoselective. Anesthesiology 83: 611–615. 7661362 10.1097/00000542-199509000-00021

[16] Hall AC, Lieb WR, Franks NP (1994) Stereoselective and non-stereoselective actions of isoflurane on the GABAA receptor. Br J Pharmacol 112: 906–910. 7921619 10.1111/j.1476-5381.1994.tb13166.x PMC1910207

[17] Franks NP, Lieb WR (1991) Stereospecific effects of inhalational general anesthetic optical isomers on nerve ion channels. Science 254: 427–430. 1925602 10.1126/science.1925602

[18] Carbo C, Yuki K, Demers M, Wagner DD, Shimaoka M (2012) Isoflurane inhibits neutrophil recruitment in the cutaneous Arthus reaction model. J Anesth 27: 261–268. 23096126 10.1007/s00540-012-1508-1 PMC3568683

[19] Shimaoka M, Xiao T, Liu JH, Yang Y, Dong Y, et al (2003) Structures of the alpha L I domain and its complex with ICAM-1 reveal a shape-shifting pathway for integrin regulation. Cell 112: 99–111. 12526797 10.1016/s0092-8674(02)01257-6 PMC4372089

[20] Yuki K, Bu W, Xi J, Sen M, Shimaoka M, et al. (2012) Isoflurane binds and stabilizes a closed conformation of the leukocyte function-associated antigen-1. FASEB J. 10.1096/fj.12-212746 PMC3475260 22815384

[21] Yuki K, Bu W, Xi J, Shimaoka M, Eckenhoff R (2013) Propofol Shares the Binding Site with Isoflurane and Sevoflurane on Leukocyte Function-Associated Antigen-1. Anesth Analg. 10.1213/ANE.0b013e3182a00ae0 PMC3844542 23960033

[22] Qu A, Leahy DJ (1996) The role of the divalent cation in the structure of the I domain from the CD11a/CD18 integrin. Structure 4: 931–942. 8805579 10.1016/s0969-2126(96)00100-1

[23] Yuki K, Soriano SG, Shimaoka M (2011) Sedative drug modulates T-cell and lymphocyte function-associated antigen-1 function. Anesth Analg 112: 830–838. 21385989 10.1213/ANE.0b013e31820dcabb PMC3073815

[24] Vincent F, Ramoni R, Spinelli S, Grolli S, Tegoni M, et al (2004) Crystal structures of bovine odorant-binding protein in complex with odorant molecules. Eur J Biochem 271: 3832–3842. 15373829 10.1111/j.1432-1033.2004.04315.x

[25] Paliwal A, De PK (2007) Purification, cloning and regulation of a novel acid-lipase-like protein of hamster expressed in lacrimal glands and tears during lactation. Biochim Biophys Acta 1771: 55–65. 17141562 10.1016/j.bbalip.2006.10.002

[26] Butts CA, Xi J, Brannigan G, Saad AA, Venkatachalan SP, et al (2009) Identification of a fluorescent general anesthetic, 1-aminoanthracene. Proc Natl Acad Sci U S A 106: 6501–6506. 19346473 10.1073/pnas.0810590106 PMC2672486

[27] Lea WA, Xi J, Jadhav A, Lu L, Austin CP, et al (2009) A high-throughput approach for identification of novel general anesthetics. PLoS One 4: e7150. 19777064 10.1371/journal.pone.0007150 PMC2746312

[28] Eckenhoff RG (2001) Promiscuous ligands and attractive cavities: how do the inhaled anesthetics work? Mol Interv 1: 258–268. 14993365

[29] Urbanova M, Dukor RK, Pancoska P, Gupta VP, Keiderling TA (1991) Comparison of alpha-lactalbumin and lysozyme using vibrational circular dichroism. Evidence for a difference in crystal and solution structures. Biochemistry 30: 10479–10485. 1931971 10.1021/bi00107a016

[30] Tanner JW, Johansson JS, Liebman PA, Eckenhoff RG (2001) Predictability of weak binding from X-ray crystallography: inhaled anesthetics and myoglobin. Biochemistry 40: 5075–5080. 11305924 10.1021/bi001428d

[31] Svergun DI, Barberato C, Koch MH, Fetler L, Vachette P (1997) Large differences are observed between the crystal and solution quaternary structures of allosteric aspartate transcarbamylase in the R state. Proteins 27: 110–117. 9037716

[32] Hodsdon ME, Cistola DP (1997) Discrete backbone disorder in the nuclear magnetic resonance structure of apo intestinal fatty acid-binding protein: implications for the mechanism of ligand entry. Biochemistry 36: 1450–1460. 9063893 10.1021/bi961890r

[33] Gaillard T, Martin E, San Sebastian E, Cossio FP, Lopez X, et al (2007) Comparative normal mode analysis of LFA-1 integrin I-domains. J Mol Biol 374: 231–249. 17919656 10.1016/j.jmb.2007.07.006

[34] Pfeiffer CC (1956) Optical isomerism and pharmacological action, a generalization. Science 124: 29–31. 13337345 10.1126/science.124.3210.29

[35] Dickinson R, White I, Lieb WR, Franks NP (2000) Stereoselective loss of righting reflex in rats by isoflurane. Anesthesiology 93: 837–843. 10969319 10.1097/00000542-200009000-00035

[36] Lysko GS, Robinson JL, Casto R, Ferrone RA (1994) The stereospecific effects of isoflurane isomers in vivo. Eur J Pharmacol 263: 25–29. 7821359 10.1016/0014-2999(94)90519-3

[37] Eger EI 2nd, Koblin DD, Laster MJ, Schurig V, Juza M, et al (1997) Minimum alveolar anesthetic concentration values for the enantiomers of isoflurane differ minimally. Anesth Analg 85: 188–192. 9212145 10.1097/00000539-199707000-00033

[38] Harris B, Moody E, Skolnick P (1992) Isoflurane anesthesia is stereoselective. Eur J Pharmacol 217: 215–216. 1425941 10.1016/0014-2999(92)90875-5

[39] Takagi J, DeBottis DP, Erickson HP, Springer TA (2002) The role of the specificity-determining loop of the integrin beta subunit I-like domain in autonomous expression, association with the alpha subunit, and ligand binding. Biochemistry 41: 4339–4347. 11914080 10.1021/bi016047u

[40] Prince JE, Brayton CF, Fossett MC, Durand JA, Kaplan SL, et al (2001) The differential roles of LFA-1 and Mac-1 in host defense against systemic infection with Streptococcus pneumoniae. J Immunol 166: 7362–7369. 11390487 10.4049/jimmunol.166.12.7362

[41] Emoto M, Emoto Y, Brinkmann V, Miyamoto M, Yoshizawa I, et al (2003) Increased resistance of LFA-1-deficient mice to lipopolysaccharide-induced shock/liver injury in the presence of TNF-alpha and IL-12 is mediated by IL-10: a novel role for LFA-1 in the regulation of the proinflammatory and anti-inflammatory cytokine balance. J Immunol 171: 584–593. 12847222 10.4049/jimmunol.171.2.584

[42] Miyamoto M, Emoto M, Emoto Y, Brinkmann V, Yoshizawa I, et al (2003) Neutrophilia in LFA-1-deficient mice confers resistance to listeriosis: possible contribution of granulocyte-colony-stimulating factor and IL-17. J Immunol 170: 5228–5234. 12734371 10.4049/jimmunol.170.10.5228

[43] Reisman NM, Floyd TL, Wagener ME, Kirk AD, Larsen CP, et al (2011) LFA-1 blockade induces effector and regulatory T-cell enrichment in lymph nodes and synergizes with CTLA-4Ig to inhibit effector function. Blood 118: 5851–5861. 21972294 10.1182/blood-2011-04-347252 PMC3228500

[44] Poston RS, Robbins RC, Chan B, Simms P, Presta L, et al (2000) Effects of humanized monoclonal antibody to rhesus CD11a in rhesus monkey cardiac allograft recipients. Transplantation 69: 2005–2013. 10852588 10.1097/00007890-200005270-00006

[45] Badell IR, Russell MC, Thompson PW, Turner AP, Weaver TA, et al (2010) LFA-1-specific therapy prolongs allograft survival in rhesus macaques. J Clin Invest 120: 4520–4531. 21099108 10.1172/JCI43895 PMC2994340

[46] Turgeon NA, Avila JG, Cano JA, Hutchinson JJ, Badell IR, et al (2010) Experience with a novel efalizumab-based immunosuppressive regimen to facilitate single donor islet cell transplantation. Am J Transplant 10: 2082–2091. 20883542 10.1111/j.1600-6143.2010.03212.x PMC3335736

[47] Boehncke WH (2007) Efalizumab in the treatment of psoriasis. Biologics 1: 301–309. 19707339 PMC2721316

[48] Graf BM, Boban M, Stowe DF, Kampine JP, Bosnjak ZJ (1994) Lack of stereospecific effects of isoflurane and desflurane isomers in isolated guinea pig hearts. Anesthesiology 81: 129–136. 8042781 10.1097/00000542-199407000-00019

[49] Moody EJ, Harris B, Hoehner P, Skolnick P (1994) Inhibition of [3H]isradipine binding to L-type calcium channels by the optical isomers of isoflurane. Lack of stereospecificity. Anesthesiology 81: 124–128. 8042780 10.1097/00000542-199407000-00018

[50] Xu Y, Tang P, Firestone L, Zhang TT (1996) 19F nuclear magnetic resonance investigation of stereoselective binding of isoflurane to bovine serum albumin. Biophys J 70: 532–538. 8770230 10.1016/S0006-3495(96)79599-1 PMC1224952

