# Supplementary figures and images for: Stereoselectivity of Isoflurane in Adhesion Molecule Leukocyte Function-Associated Antigen-1
Source: PLoS One. 2014 May 6;9(5):e96649. doi: 10.1371/journal.pone.0096649 (PMC4011845; doi:10.1371/journal.pone.0096649)

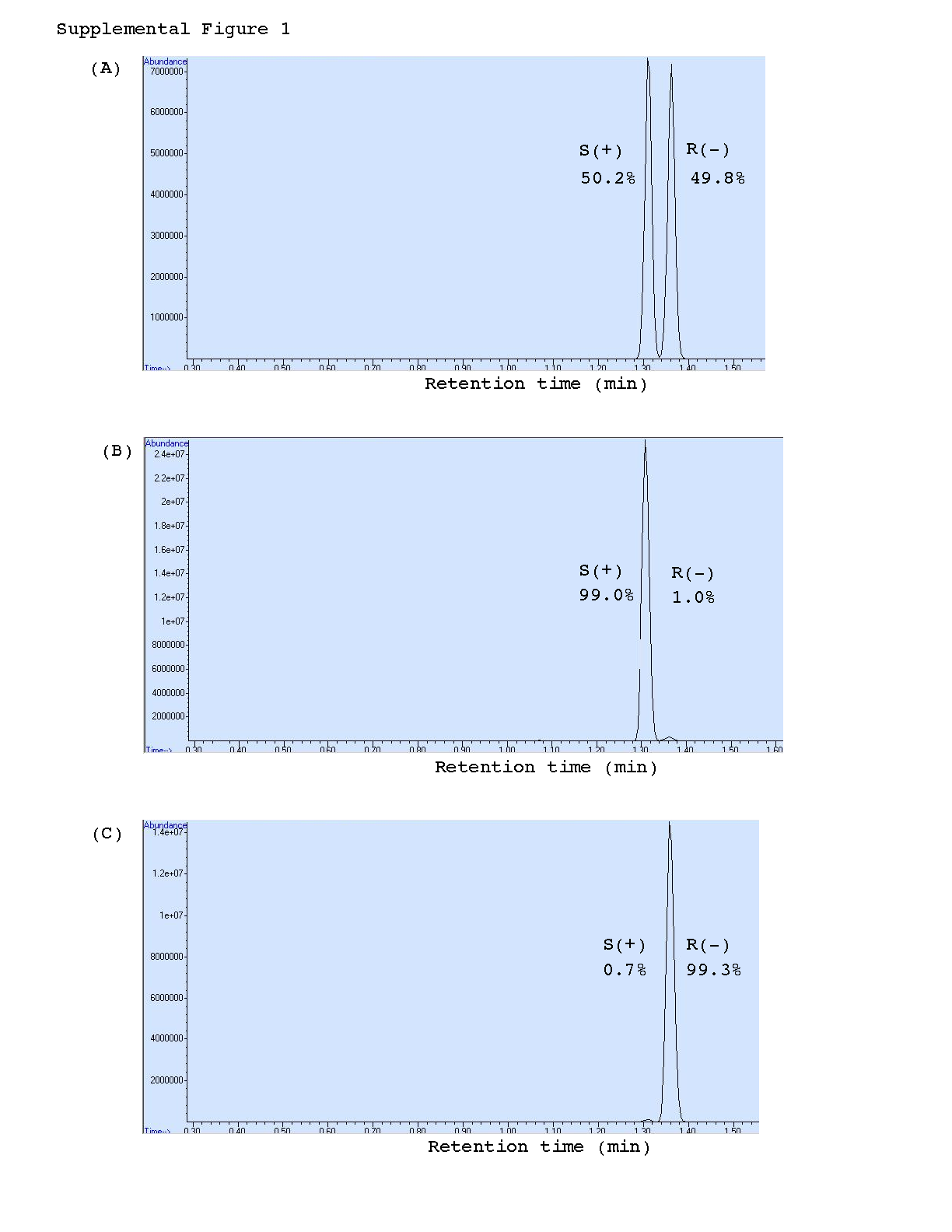

Supplement: Figure S1 — Chiral gas chromatography of isoflurane. The gas chromatographic traces were shown. (A) racemic isoflurane, (B) S- or (C) R- isoflurane. The area under the curve was used to calculate the composition of S- and R-isoflurane in each solution. X-axis represents retention time (min), and y-axis represents detector (arbitrary unit). (TIFF) [file pone.0096649.s001.tif]
